# Gastric Necrosis Due to Small Bowel Obstruction: A Case Report

**DOI:** 10.7759/cureus.37936

**Published:** 2023-04-21

**Authors:** Fernando A Nuñez-Moreno, Lissvia E Acosta-Gaxiola

**Affiliations:** 1 Gastrointestinal Surgery, La Unidad Médica de Alta Especialidad (UMAE) Hospital de Especialidades, Mexico City, MEX; 2 General Practice, Instituto Mexicano del Seguro Social Hospital General Regional No. 1, Tijuana Baja California, MEX

**Keywords:** massive gastro, alzheimer’s dementia, general emergency surgery, femoral hernia, gastric body necrosis

## Abstract

Gastric necrosis is a rare entity due to the normal anatomy of the stomach; the irrigation of the stomach is abundant and has vast collateral irrigation that prevents necrosis from happening in normal situations. Gastric ischemia doesn’t happen even if arterial occlusion occurs; however, a venous occlusion caused by an increase in intragastric pressure (measured ﻿>20 cm H2O in some experiments) that surpasses gastric venous pressure can precipitate necrosis of the stomach. Here we present the case of a 79-year-old woman with a history of chronic smoking, Alzheimer’s dementia, systemic hypertension, hypothyroidism, chronic constipation, and a hysterectomy performed 25 years ago. An exploratory laparotomy was performed with the following findings: 3 liters of fecaloid fluid in the abdominal cavity, 70% necrosis of the stomach affecting major curvature and 80% of the fundus without compromising the cardia, a perforation in the anterior portion of the stomach with a diameter of 6 cm, a right femoral hernia with small bowel entrapment, intestinal obstruction with dilated small bowel; and intestinal necrosis of 7 cm of the ileum that was inside the femoral hernia. A vertical gastrectomy for the necrotic stomach and intestinal resection with termino-terminal anastomosis in the affected segment of the ileum were performed. The patient had a poor response to treatment and finally died 72 hours after surgery due to abdominal sepsis. This report shows that gastric necrosis, although rare, can be a cause of acute abdominal pain. It highlights the importance of a good clinical examination and imaging studies in detecting the causes of small bowel obstruction and offering prompt diagnosis and treatment to patients with small bowel obstruction.

## Introduction

Gastric necrosis is a rare condition that usually does not occur due to the abundant irrigation and collateral circulation of the stomach [1]. In fact, even ligation of major vessels does not typically lead to gastric necrosis [2].

While arterial occlusion does not usually cause gastric ischemia, venous occlusion due to increased intragastric pressure (measured at >20 cm H2O in some studies) that exceeds gastric venous pressure can lead to gastric necrosis [3,4].

Gastric necrosis has been reported in various clinical scenarios, including gastrostomy prolapse, gastric banding surgery, inguinal hernia with entrapment of gastric walls, the rapid expansion of the stomach following a large meal, and patients who have undergone fundoplication surgery [1,2,5-7].

## Case presentation

We present the case of a 79-year-old woman with a medical history of chronic smoking, Alzheimer's dementia, systemic hypertension, hypothyroidism, chronic constipation, and a hysterectomy performed 25 years ago.

The patient experienced diffuse abdominal colicky pain and constipation for four days before seeking medical attention at a local clinic. She was treated with prokinetics without improvement and subsequently underwent a second medical assessment. During the examination, the patient exhibited a temperature of 37.1°C, a pulse rate of 110 beats per minute, 22 breaths per minute, and a blood pressure of 90/40 mmHg. There were also signs of dehydration, altered mental status, and metabolic acidosis. A nasogastric tube was inserted, and four liters of fecaloid matter were obtained. After fluid resuscitation, the patient was transferred to our hospital's emergency department, where she presented with a temperature of 37.6°C, a pulse rate of 80 beats per minute, 18 breaths per minute, a blood pressure of 110/70 mmHg, and a normal mental status. During the surgical assessment, the patient did not report any abdominal or groin pain, and the physical examination did not reveal any evidence of hernias or signs indicating a surgical emergency.

Initial blood analysis showed a mild electrolyte imbalance and elevation of creatinine, indicating dehydration, which was associated with vomiting (Table 1).

**Table 1:** Initial blood analysis

Laboratory parameter	Values	Normal range
Glucose	97 mg/dL	70-110 mg/dL
Creatinine	2.45 mg/dL	0.7-1.3 mg/dL
Sodium (Na)	138 mEq/L	135-145 mEq/L
Potassium (K)	4.6 mEq/L	3.5-5 mEq/L
Chloride (Cl)	103 mEq/L	95-105 mEq/L
White blood cell (WBC)	8.6 cel/mm3	5,000-10,000 cel/mm3
Hemoglobin (Hb)	16.5 mg/dL	13.5-17.5 mg/dL
Platelet (Plt)	199,000 cel/mm3	150,000-450,000 cel/mm3
Lactate	1.1 mmol/L	0.5-1.0 mmol/L

Because the patient did not exhibit any signs of peritonitis, no hernias were detected during the physical examination, and the patient had a history of abdominal surgery, this led to the suspicion that the obstruction may be related to the adhesive disease. 

The patient received conservative management with nasogastric tube decompression, fluid resuscitation, ceftriaxone 1g twice daily, metronidazole 500 mg three times daily, and close clinical observation.

**Figure 1:**
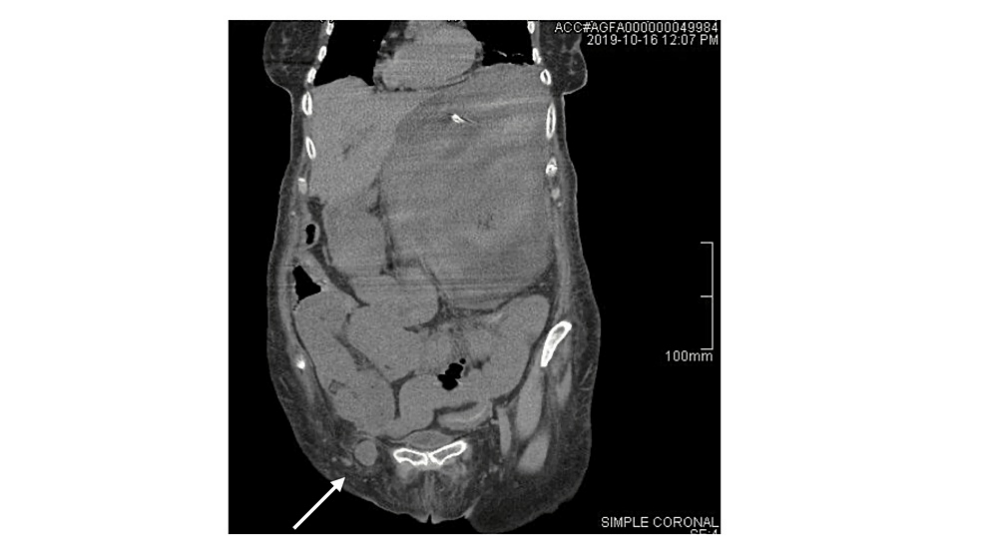
A coronal section of an abdominal CT scan that shows a right femoral hernia containing the small bowel

However, due to an unsatisfactory evolution (the patient continued to have high output from the nasogastric tube, persistent abdominal pain, and no passage of the flatus) 12 hours after the initial assessment, mechanical obstruction was suspected, and a CT scan with intravenous contrast was performed, revealing gastromegaly and a right femoral hernia with intestinal content (Figure 1).

The patient and her family were informed about the patient's condition, which included a small bowel obstruction and a femoral hernia that required surgical intervention.

**Figure 2:**
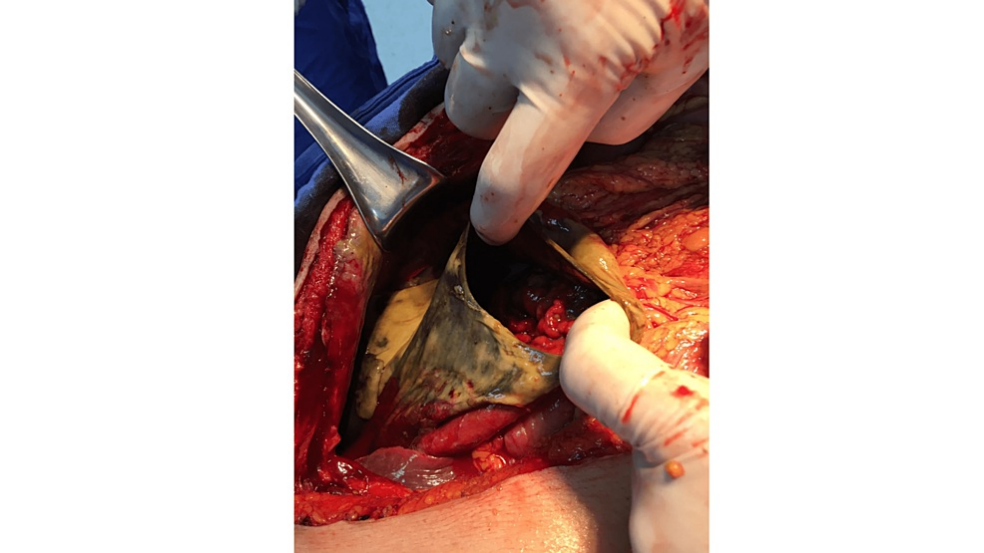
Gastric wall necrosis and perforation found during laparotomy

The patient underwent an exploratory laparotomy 14 hours after initial assessment, during which the following findings were observed: a significant amount of fecaloid fluid, approximately three liters, was found in the abdominal cavity. Additionally, there was 70% necrosis of the stomach, affecting the major curvature, and 80% of the fundus without compromising the cardia. A perforation with a diameter of 6 cm was also observed in the anterior portion of the stomach (Figure 2). In addition to these findings, a right femoral hernia with small bowel entrapment was identified, which caused intestinal obstruction. Moreover, the small bowel was dilated, and a 7-cm section of the ileum inside the femoral hernia was necrotic.

**Figure 3:**
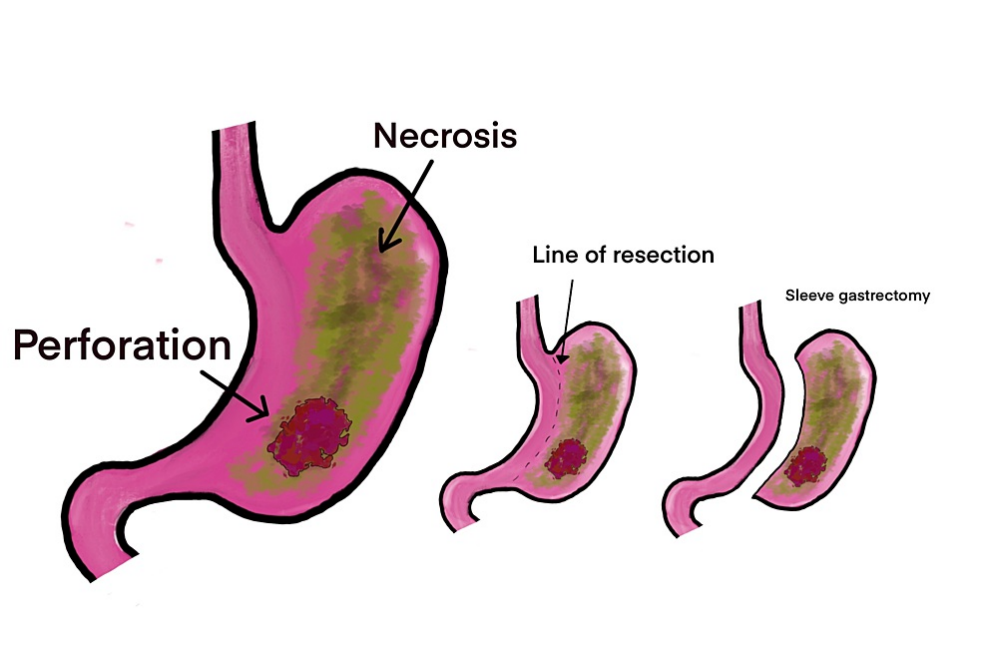
Depiction of gastric necrosis, perforation, and the vertical gastrectomy performed to resect nonviable tissue

Abundant irrigation was performed with sterile saline solution before repairing the femoral hernia using the preperitoneal technique of Lloyd M. Nyhus with Vycril 3-0. Also, a vertical gastrectomy was performed to remove the necrotic portion of the stomach, followed by an intestinal resection with a termino-terminal manual anastomosis using Vycril 3-0 in two layers in the affected segment of the ileum (Figure 3).

In the postoperative period, the patient required endotracheal intubation and vasopressor support with norepinephrine. Broad-spectrum antibiotics, including meropenem, were used. Despite the treatment provided, the patient exhibited a poor response and ultimately passed away 72 hours after the surgery due to abdominal sepsis.

## Discussion

This report highlights a rare occurrence of gastric necrosis resulting from bowel and gastric dilatation caused by a femoral hernia. The severe gastric dilatation was likely due to small bowel obstruction, which eventually surpassed the venous pressure of the gastric walls, leading to ischemia, necrosis, and stomach perforation. The mechanism of acute gastric dilatation and gastric ischemia has been explained in detail, stating that sympathetic nerve stimulation diminishes gastric tone, leading to relaxation of the gastric corpus and an increase in pyloric sphincter tone. This perpetuates gastric dilatation until it surpasses the venous pressure, causing ischemia of the gastric walls [8]. Just 3 liters of fluid can distend a normal stomach to this point of tension, and with 4 liters of fluid, intragastric pressure of 120 to 150 mmHg can occur, resulting in gastric rupture [6]. Furthermore, after dilatation of the gastric corpus, the angle between the esophagus and the right crus of the esophageal hiatus becomes more acute, and the esophagogastric junction functions as a one-way valve, leading to obstruction of the gastroesophageal junction [2]. This can explain why the patient could not release gastric content by vomiting, thus avoiding gastric dilatation and necrosis.

Gastric necrosis can result from various causes, including overeating, direct damage to gastric walls, and gastric prolapse through a gastrostomy [1,2,4,5].

During laparotomy, the patient had a perforation of the gastric walls, which was previously reported in a 13-year-old girl who presented with acute infarction of the stomach secondary to copious food ingestion [9]. Their findings included a free perforation associated with necrosis of the stomach, similar to our patient’s.

The case report emphasizes the importance of prompt diagnosis and early treatment of gastric necrosis, as it is a life-threatening condition with a high mortality rate. As shown in this case, physical examination alone was not enough to detect the presence of a small femoral hernia or signs of intestinal ischemia or peritonitis. This could be due to the patient's cognitive impairment. Although a proper clinical examination is crucial for detecting hernias as a cause of SBO, it may not be sufficient in patients who are elderly or have cognitive impairment. In such cases, a CT scan can provide more comprehensive information and aid in prompt surgical decision-making. Delayed diagnosis can have devastating consequences, making timely diagnosis and management essential. However, in places with limited resources, conducting a CT scan may not always be feasible, posing a challenge to SBO diagnosis and management. 

## Conclusions

Gastric necrosis is a life-threatening condition that should be considered a potential cause of acute abdominal pain, particularly in patients with cognitive impairment. In such cases, a physical examination may be unreliable, highlighting the importance of imaging tests for an accurate diagnosis. Given its high mortality rate, prompt recognition and management of gastric necrosis are critical. In hospitals with limited resources, imaging studies may be limited, underscoring the importance of clinical examination to detect causes of small bowel obstruction that require prompt surgical management. Overall, the report adds to the existing knowledge of the causes and mechanisms of gastric necrosis.
